# Highly pathogenic avian influenza H5N1 virus infections in pinnipeds and seabirds in Uruguay: Implications for bird–mammal transmission in South America

**DOI:** 10.1093/ve/veae031

**Published:** 2024-04-13

**Authors:** Gonzalo Tomás, Ana Marandino, Yanina Panzera, Sirley Rodríguez, Gabriel Luz Wallau, Filipe Zimmer Dezordi, Ramiro Pérez, Lucía Bassetti, Raúl Negro, Joaquín Williman, Valeria Uriarte, Fabiana Grazioli, Carmen Leizagoyen, Sabrina Riverón, Jaime Coronel, Soledad Bello, Enrique Páez, Martín Lima, Virginia Méndez, Ruben Pérez

**Affiliations:** Sección Genética Evolutiva, Facultad de Ciencias, Universidad de la República, Iguá 4225, Montevideo 11400, Uruguay; Sección Genética Evolutiva, Facultad de Ciencias, Universidad de la República, Iguá 4225, Montevideo 11400, Uruguay; Sección Genética Evolutiva, Facultad de Ciencias, Universidad de la República, Iguá 4225, Montevideo 11400, Uruguay; Departamento de Virología, División de Laboratorios Veterinarios ‘Miguel C. Rubino’, Dirección’General de Servicios Ganaderos, Ministerio de Ganadería, Agricultura y Pesca, Ruta 8 km 17,000, Montevideo 12100, Uruguay; Departamento de Entomología, Núcleo de Bioinformática, Instituto Aggeu Magalhães (IAM)-Fundação Oswaldo Cruz (FIOCRUZ), Av. Moraes Rego, s/n, Campus da UFPE- Cidade Universitária, Recife, Pernambuco 50740-465, Brazil; Department of Arbovirology and Entomology, Bernhard Nocht Institute for Tropical Medicine, WHO Collaborating Center for Arbovirus and Hemorrhagic Fever Reference and Research, National Reference Center for Tropical Infectious Diseases, Hamburg 20359, Germany; Departamento de Entomología, Núcleo de Bioinformática, Instituto Aggeu Magalhães (IAM)-Fundação Oswaldo Cruz (FIOCRUZ), Av. Moraes Rego, s/n, Campus da UFPE- Cidade Universitária, Recife, Pernambuco 50740-465, Brazil; Departamento de Virología, División de Laboratorios Veterinarios ‘Miguel C. Rubino’, Dirección’General de Servicios Ganaderos, Ministerio de Ganadería, Agricultura y Pesca, Ruta 8 km 17,000, Montevideo 12100, Uruguay; Departamento de Virología, División de Laboratorios Veterinarios ‘Miguel C. Rubino’, Dirección’General de Servicios Ganaderos, Ministerio de Ganadería, Agricultura y Pesca, Ruta 8 km 17,000, Montevideo 12100, Uruguay; Departamento de Virología, División de Laboratorios Veterinarios ‘Miguel C. Rubino’, Dirección’General de Servicios Ganaderos, Ministerio de Ganadería, Agricultura y Pesca, Ruta 8 km 17,000, Montevideo 12100, Uruguay; Sección Genética Evolutiva, Facultad de Ciencias, Universidad de la República, Iguá 4225, Montevideo 11400, Uruguay; Dirección Nacional de Biodiversidad y Servicios Ecosistémicos (DINABISE), Ministerio de Ambiente, Juncal 1385, Montevideo 11000, Uruguay; Dirección Nacional de Biodiversidad y Servicios Ecosistémicos (DINABISE), Ministerio de Ambiente, Juncal 1385, Montevideo 11000, Uruguay; Dirección Nacional de Biodiversidad y Servicios Ecosistémicos (DINABISE), Ministerio de Ambiente, Juncal 1385, Montevideo 11000, Uruguay; Dirección Nacional de Recursos Acuáticos (DINARA), Ministerio de Ganadería, Agricultura y Pesca, Constituyente 1497, Montevideo 11200, Uruguay; Dirección Nacional de Recursos Acuáticos (DINARA), Ministerio de Ganadería, Agricultura y Pesca, Constituyente 1497, Montevideo 11200, Uruguay; Dirección Nacional de Recursos Acuáticos (DINARA), Ministerio de Ganadería, Agricultura y Pesca, Constituyente 1497, Montevideo 11200, Uruguay; Dirección Nacional de Recursos Acuáticos (DINARA), Ministerio de Ganadería, Agricultura y Pesca, Constituyente 1497, Montevideo 11200, Uruguay; Dirección Nacional de Recursos Acuáticos (DINARA), Ministerio de Ganadería, Agricultura y Pesca, Constituyente 1497, Montevideo 11200, Uruguay; Dirección Nacional de Recursos Acuáticos (DINARA), Ministerio de Ganadería, Agricultura y Pesca, Constituyente 1497, Montevideo 11200, Uruguay; Sección Genética Evolutiva, Facultad de Ciencias, Universidad de la República, Iguá 4225, Montevideo 11400, Uruguay

**Keywords:** avian influenza, mammal adaptation, South America, sea mammals, transmission

## Abstract

The highly pathogenic avian influenza viruses of clade 2.3.4.4b have caused unprecedented deaths in South American wild birds, poultry, and marine mammals. In September 2023, pinnipeds and seabirds appeared dead on the Uruguayan Atlantic coast. Sixteen influenza virus strains were characterized by real-time reverse transcription PCR and genome sequencing in samples from sea lions (*Otaria flavescens*), fur seals (*Arctocephalus australis*), and terns (*Sterna hirundinacea*). Phylogenetic and ancestral reconstruction analysis showed that these strains have pinnipeds most likely as the ancestral host, representing a recent introduction of clade 2.3.4.4b in Uruguay. The Uruguayan and closely related strains from Peru (sea lions) and Chile (sea lions and a human case) carry mammalian adaptative residues 591K and 701N in the viral polymerase basic protein 2 (PB2). Our findings suggest that clade 2.3.4.4b strains in South America may have spread from mammals to mammals and seabirds, revealing a new transmission route.

## Introduction

The *Alphainfluenzavirus influenzae* species (family *Orthomyxoviridae*) is a prominent pathogen in birds and mammals. The virus is divided into subtypes based on the genetic and antigenic properties of hemagglutinin (HA) and neuraminidase (Mostafa et al. 2018).

Migratory wild birds, especially waterfowl, are the natural host and reservoir of the avian influenza virus (AIV) and harbor most combinations of circulating subtypes (Spackman 2008). Influenza in mammals evolved from the adaptation of ancestral avian viruses. The remarkable ability of the influenza virus to adapt to new hosts is associated with its segmented negative single-stranded RNA genome, which mutates and reassorts easily (Mostafa et al. 2018).

Low-pathogenicity AIV (LPAIV) of the H5 and H7 subtypes may evolve toward highly pathogenic AIV (HPAIV) upon transmission into highly dense domestic bird populations (Lee, Criado, and Swayne 2021). HPAIV strains are primarily characterized by multiple basic amino acid residues at the HA cleavage site processed by ubiquitous proteases, resulting in a fast-spreading deadly disease with increased pathogenicity (de Bruin et al. 2022).

Since October 2020, HPAIV clade 2.3.4.4b (H5N1) from the Goose/Guangdong (Gs/GD) lineage has been responsible for an unprecedented number of deaths in wild birds and poultry (Cui et al. 2022). Clade 2.3.4.4b spread from Asia to Africa and Europe and reached North America via Iceland (Günther et al. 2022). The first detections of HPAIV clade 2.3.4.4b in North America occurred in wild and domestic birds in November 2021 in Canada and late December 2021 in the USA (Bevins et al. 2022; Caliendo et al. 2022; Alkie et al. 2023). In October 2022, it emerged in South America, with severe outbreaks identified in Argentina, Bolivia, Brazil, Chile, Colombia, Ecuador, Peru, Uruguay, and Venezuela (Adlhoch et al. 2023a; Leguia et al. 2023; Marandino et al. 2023; Reischak et al. 2023).

There have been increasing reports of deadly outbreaks among wild and captive mammals caused by clade 2.3.4.4b. Both terrestrial and marine mammals have been affected, including farmed minks (*Neovison vison*) in Spain, true seals (*Phocidae*) in the USA, and sea lions (*Otaria flavescens/byronia*) in Peru and Chile (Agüero et al. 2023; Leguia et al. 2023; Pardo-Roa et al. 2023; Puryear et al. 2023; Ulloa et al. 2023). Infections in companion animals, such as household cats and dogs, have been reported in Italy, France, Poland, and the USA (Briand et al. 2023; Moreno et al. 2023; Rabalski et al. 2023; Sillman et al. 2023; Ly 2024).

The high frequency of HPAIV spillover into mammals, including humans, raises serious concerns about a potential global pandemic as the virus could adapt and transmit among mammals. However, reporting HPAIV H5N1 in mammals was primarily associated with bird-to-mammal transmission (Rabalski et al. 2023).

Marine mammals seem particularly susceptible to avian influenza. More than 15,000 infected sea lions in Peru and Chile died between November 2022 and June 2023 (Leguia et al. 2023; Pardo-Roa et al. 2023; Ulloa et al. 2023). The southernmost case of H5N1 on the Pacific coast was detected in June 2023 in a sea lion from Puerto Williams, Chile. The first infected sea lions on the Atlantic coast were reported in the southernmost tip of Argentina (Rio Grande, Tierra del Fuego Province) in August 2023 (Adlhoch et al. 2023b).

The present study conducted viral detection and genome sequencing on infected sea lions (*Otaria flavescens*), fur seals (*Arctocephalus australis*), and sea birds (*Sterna hirundinacea)* sampled in Uruguay from September to October 2023. Our findings suggest mammal-to-mammal transmission and bird infection by viruses of mammalian origin.

## Materials and methods

### AIV detection and HA subtyping

During September and early October 2023, ocular, oropharyngeal, encephalic, rectal/cloacal, and fecal swabs were obtained from pinnipeds and seabirds from different regions of the Uruguayan coastline. Samples were collected and promptly transported for molecular diagnosis (Marandino et al. 2023). Clinical signs could not be determined in deceased animals. Carcasses were buried immediately according to sanitary protocol, precluding autopsy.

Viral detection and HA subtyping were performed by real-time reverse transcription PCR, as previously described (Marandino et al. 2023).

### Illumina sequencing

Complementary DNA (cDNA) was synthesized using the Superscript II First-Strand Synthesis System (Thermo Fisher Scientific, Waltham, MA, USA). Full AIV segment amplification was performed in a single reaction with a primer pair (Zhou et al. 2009) or using unique primers for each viral segment (Hoffmann et al. 2001). Amplicons were pooled and purified using AMPure XP (Beckman Coulter, Indianapolis, IN, USA), and 100 ng was subjected to the Nextera™ DNA Flex Library Preparation kit (Illumina, San Diego, CA, USA) employing a unique dual indexing (XT-Index Kit V2-Set A). Libraries Quantification and sequencing were performed as previously reported (Marandino et al. 2023).

### Genome analysis

The raw-sequencing data were trimmed and filtered using BBDuk and Minimap2 plugins in Geneious (Kearse et al. 2012). The clean reads were mapped to a South American avian influenza genome (GenBank accessions: OQ547340–OQ547347). Assemblies were visually inspected and optimized; annotations were transferred from reference strains and manually curated. Recombinant segments were evaluated with the complete genome datasets using the RDP4 v4.95 software (Martin et al. 2015).

Intra-host variants were analyzed using Geneious by assembling clean reads to obtain consensus and recording minor single-nucleotide variants (SNVs) with a frequency greater than 10 percent and a minimum coverage depth of 100.

### Dataset

We downloaded all sequences from Ecuador, Peru, Chile, Argentina, and Uruguay from GISAID (https://gisaid.org/) (7 October 2023). The USA strain (A/fox/Minnesota/22/016487/001|EPI_ISL_15078249) was included as an outgroup. Sequences were aligned using MAFFT v 7 (Katoh and Standley 2013) and trimmed to each segment’s starting ATG and ending STOP codon. Maximum-likelihood phylogenetic trees were obtained using FastTree (Price et al. 2009) and IQTREE 2.2.0 (Minh et al. 2020) with approximate likelihood ratio tests (aLRT) for internal node support.

### Molecular clock and ancestral state reconstruction

Concatenated segments were used to reconstruct the phylogenetic trees. The root-to-tip correlation found in the best ML tree recovered by IQTREE was *R* = 0.909 based on TempEst v 1.5.3 (Rambaut et al. 2016). The timetree reconstruction was performed using the uncorrelated molecular clock, and three coalescent models were tested using the path sampling analysis (constant size, exponential growth, and Bayesian skygrid). The nucleotide substitution model was set as GTR + G + I, based on the best model found by ModelFinder from IQTREE 2.2.0 in Beast 1.10.5 (Suchard et al. 2018). Base frequencies were estimated from the alignment, and four gamma categories were added as parameters. The uncorrelated molecular clock model and Bayesian skygrid coalescent model output the best likelihood results, which were selected for further analysis. Efective Sample Size (ESS) values were all above 200 for the tested runs.

Bayesian phylogenetic reconstruction was performed using the BEAST 1.10.5 software. The analysis utilized stamped tip dates information and three categorical data for discrete phylogeographic analysis: country, host, and the presence of critical amino acid substitutions. We set up 500 million chains and sampled every 50,000 trees for enough parameter mixing and estimates. ESS values were above 200, validating the optimal parameter space exploration. Burning was set to 10 per cent for all trees generated. Finally, a phylogenetic tree was depicted with Figtree. We obtained silhouette images using PHYLOPIC (https://www.phylopic.org/).

### Map and case dynamics through time visualization

To display the distribution of H5N1 HPAI cases in South America associated with or not associated with genomic sequences, we downloaded the dataset from https://empres-i.apps.fao.org/. We set up Microreact instances harboring the whole dataset from South America (starting on Jan 1^st^, 2023) of FAO-confirmed H5N1 cases (https://microreact.org/project/vBkfwLENtQqwJ5G7YZfZDb-influenzasea-lionsacases) (Argimón et al. 2016).

### Augur and individual ML tree display

TreeTime was employed to reconstruct ancestral sequences using a maximum-likelihood approach and to map nucleotide mutations for each branch in the phylogenetic tree compared with the reference sequence A/Goose/Guangdong/1/96 (H5N1) (Sagulenko, Puller, and Neher 2018).

## Results

### HPAIV H5N1 outbreak in Uruguay

Out of ninety samples (seventy-two pinnipeds and eighteen seabirds) analyzed until 4 October, twenty-nine pinnipeds and four terns tested positive for HPAIV H5N1 (Supplementary Table S1). Samples for different locations with lower threshold cycles (*C_t_*) were selected for genome sequencing (Table 1).

**Table 1.** Samples used in the study.

Isolate (influenza type/host/country/strain ID/year)	Date	Location	Swab^a^	*C_t_* ^b^	Host	Symptoms/age/sex
A/sea lion/Uruguay/P4_6923/2023	6 Sep 2023	Maldonado (Punta del Este)	B	23.61	*Otaria flavescens*	Dead/adult
A/sea lion/Uruguay/P5_6923/2023	6 Sep 2023	Canelones (El Pinar)	N	29.24	*Otaria flavescens*	Dead
A/sea lion/Uruguay/P6_6923/2023	6 Sep 2023	Canelones (El Pinar)	O	28.1	*Otaria flavescens*	Neurological
A/sea lion/Uruguay/P7_6923/2023	6 Sep 2023	Canelones (Médanos de Solymar)	CSF	28.1	*Otaria flavescens*	Dead
A/fur seal/Uruguay/P8_8923/2023	8 Sep 2023	Rocha (La Paloma)	*OP*	31.5	*Arctocephalus australis*	NA
A/sea lion/Uruguay/P10_8923/2023	8 Sep 2023	Rocha (Cabo Polonio)	*OP/R*	25.1	*Otaria flavescens*	NA
A/sea lion/Uruguay/P13_11923/2023	11 Sep 2023	Canelones (Atlántida)	*N*	22.9	*Otaria flavescens*	Dead/M
A/sea lion/Uruguay/P14_11923/2023	11 Sep 2023	Canelones (Parque del Plata)	*N/R*	23.5	*Otaria flavescens*	Dead/M
A/sea lion/Uruguay/P15_14923/2023	14 Sep 2023	Isla de Flores	R	29.3	*Otaria flavescens*	Male/juvenile
A/tern/Uruguay/P16_14923/2023	14 Sep 2023	Isla de Flores	*O/C*	20.2	*Sterna hirundinacea*	Dead/M/juvenile
A/sea lion/Uruguay/P17_14923/2023	14 Sep 2023	Isla de Lobos	*N*	25.5	*Otaria flavescens*	Dead
A/sea lion/Uruguay/P18_14923/2023	14 Sep 2023	Rocha (Aguas Dulce)	*N*	23.8	*Otaria flavescens*	Dead/male
A/tern/Uruguay/P23_41023/2023	4 Oct 2023	Rocha (Cabo Polonio)	*OP/C*	21.7	*Sterna hirundinacea*	Neurological
A/tern/Uruguay/P24_41023/2023	4 Oct 2023	Rocha (Cabo Polonio)	*OP/C*	28.6	*Sterna hirundinacea*	Dead
A/tern/Uruguay/P25_41023/2023	4 Oct 2023	Rocha (Cabo Polonio)	*OP*	24.5	*Sterna hirundinacea*	Neurological
A/fur seal/Uruguay/P26_21023/2023	2 Oct 2023	Rocha (Cabo Polonio)	*N*	24.4	*Arctocephalus australis*	Dead/adult/F

a Types of samples: N: nasal; OP: oropharyngeal; C: cloacal; R: rectal; O: ocular; B; brain; CSF: cerebrospinal fluid.

b *C_t_* in the real-time reverse transcription PCR for the matrix gene.

### Genome variability and comparison

We obtained sixteen viral genomes from ten sea lions (*Otaria flavescens*), two fur seals (*Arctocephalus australis*), and four terns (*Sterna hirundinacea*) (Table 1). Seven pinnipeds and three terns yielded complete viral genome sequences (Supplementary Table S2). For the remaining samples (*n* = 6), partial sequences comprising at least six genome segments were obtained. All the sequences were deposited in the GenBank database with accession numbers OR912247–OR912366.

The complete coding genome variability was low (distance > 99.8 per cent), and the segment sequences obtained showed the highest BLAST similarity, up to 100 per cent similarity, with strains of the HPAI A/H5N1 2.3.4.4b lineage obtained from avian and mammalian hosts in South America.

### Phylogenetic analysis

Phylogenetic analyses were performed individually on the eight genome segments (Supplementary Fig. S1). Individual phylogenetic trees show similar relationships for the Uruguayan sequences; they were closely related and associated with Peruvian and Chilean strains. These strains belonged to the B2.2 genotype according to the classification performed in North American strains (Youk et al. 2023). This genotype has segments derived from both North American (PB2, PB1, NP, and NS) and Eurasian (PA, HA, NA, and M) lineages (Agüero et al. 2023; Leguia et al. 2023; Marandino et al. 2023; Pardo-Roa et al. 2023; Youk et al. 2023).

**Figure 1.**
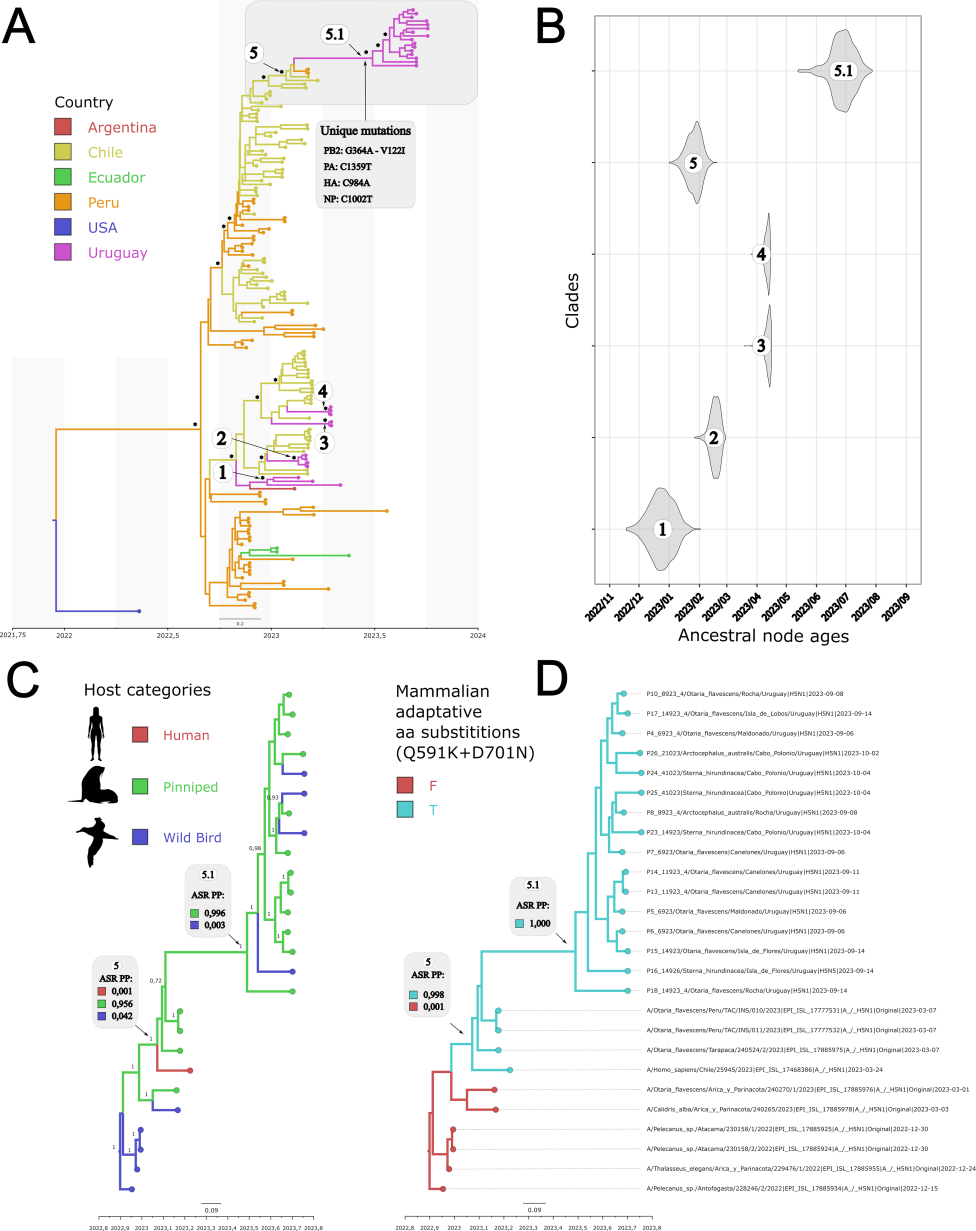
Bayesian phylogenetic reconstruction of concatenated segments, including timing estimates of the most recent common ancestors of specific Uruguayan clades and ancestral state reconstruction of countries, hosts, and presence/absence of two PB2 mutations known to increase the infection in mammalian cells. (A) Discrete phylogeographic analysis of South American countries. Country colors denote specific countries, asterisks are posterior probability support above 90, and numbers indicate distinct Uruguayan clades. (B) tMRCA of the six selected clades highlighted in (A). (C) Discrete ancestral host reconstruction and associated ancestral state reconstruction posterior probabilities of each trait (ASR PP). (D) Discrete ancestral reconstruction of PB2 mammal adaptation double mutation and associated ASR PP values.

The South American dataset showed low-sequence variability, with fewer informative phylogenetic sites per segment. We concatenated all coding sequences (CDS) for the genomes to augment the phylogenetic signal. The Uruguayan sequences from sea lions, fur seals, and terns were clustered in a monophyletic clade, denoted clade 5.1 (Fig. 1A), with the highest posterior probability [PP] support of 1 (containing four unique nucleotide substitutions and one amino acid change). The 5.1 clade tMRCA was estimated in late June 2023 (95 per cent High Probability Density [HPD] between early June and late July 2023) (Fig. 1A and B—clade 5.1). This clade also clustered with high support (PP =1) to sequences from Peru (sea lions) and Chile (sea lions and a human case) in March 2023 (Fig. 1A—clade 5). Clade 5 tMRCA dates to early January 2023, with 95 per cent HPD between late December 2022 and late February 2023. A sanderling (*Calidris alba*) and a sea lion strain also sampled in Chile were basal to this clade 5 (PP = 1).

The HPAIV strains of the February–May 2023 Uruguayan outbreaks, which affected wild birds and backyard poultry, are phylogenetically distant and did not form a monophyletic group with clade 5.1. They are associated with strains from Argentina, Peru, and Chile (Fig. 1A and B—clades 1–4).

### Mammalian adaptive markers

Viruses from the Uruguayan pinnipeds and the four South American terns have the Q591K and D701N amino acid substitutions in PB2 (Fig. 1, Supplementary Table S3). These substitutions occurred in only a few other South American strains: two strains from Peruvian sea lions, one from a Chilean sea lion, and one from a Chilean human case (Fig. 1A—clade 5) (Supplementary Table S4). All these viruses from Chile, Peru, and Uruguay, with 591K and 701N residues, form a monophyletic group (PP =1) with pinnipeds as the ancestral host (Fig. 1C—clade 5) showing an ancestral state reconstruction posterior probability of 0.956.

Two strains basal to clade 5, obtained from a sanderling, *C. alba* (EPI_ISL_17885978), and a sea lion (EPI_ISL_17885976) from Chile, have 701N but not 591K (Supplementary Table S4). In this case, the ancestral host reconstruction showed a similar probability of 48 and 51 per cent for avian and pinniped, respectively. The 701N and 591K residues in the PB2 segment were not observed for all remaining viral genomes analyzed.

Uruguayan samples belonging to clade 5.1 also depicted the substitutions PB2-V122I and PA-E237A (except for one virus), which were absent in any Chilean or Peruvian virus (Supplementary Table S4).

### Genomic epidemiology

We reconstructed the phylogeographic dispersion of the H5N1 lineage in South America using discrete and continuous phylogeographic models. However, the phylogeographic reconstruction was impacted due to the almost complete lack of sequences from Southern Chile, Argentina, Bolivia, and Paraguay. The adjusted robust discrete phylogeographic analysis, considering the dataset’s large sampling gap, indicates that Uruguayan sequences from sea lions, fur seals, and terns likely originated from Chile (ASR PP = 0.9983).

Even though there are substantial sampling gaps in genomic surveillance in South America, there is consistent diagnostic data reported by FAO (https://empres-i.apps.fao.org/). Therefore, to visualize the case counts based on the larger animal categories (wild birds, pinnipeds, terrestrial mammals, and humans), we displayed all cases into two Microreact instances. One includes the concatenated maximum-likelihood phylogenetic tree depicting it on the South American map and associated metadata (https://microreact.org/project/nSinj3LbMRme4gqussrTaU-influenzasea-lionurug). The second consists of all confirmed cases cataloged by FAO in South America after 1 January 2023 (https://microreact.org/project/vBkfwLENtQqwJ5G7YZfZDb-influenzasea-lionsacases). Cases until 31 July 2023 showed an initial coastal and an internal spreading involving wild birds, backyard poultry, and outbreaks in commercial farms. After the first cases of pinnipeds in Peru, the virus spread to the South of the Pacific coast (Supplementary Fig. S2). From 1 August, registers appeared south of the Atlantic coast and spread northward, with fewer cases involving birds and case detections in the interior of South America (Supplementary Fig. S2).

### Intra-host variability of the sequenced genomes

Intra-host variation was observed within the virus segments in some samples, except for the PB1 segment. These variations were in single-nucleotide changes that sometimes affected the amino acid sequences (Supplementary Table S5). Apart from a single nonsynonymous substitution in the M gene (H319N), minor variants were unique to each strain (i.e. they were not present in multiple sequences).

## Discussion

From September to October 2023, HPAIV subtype H5N1 caused the death of pinnipeds (*Otaria flavescens* and *Arctocephalus australis*) along 270 km of the Uruguayan coastline. Some terns (*Sterna hirundinacea*) were also found dead or with neurological symptoms a month after the first pinniped detections (Table 1, Supplementary Table S1).

The estimated population size of the sea lion *Otaria flavescens* in Uruguay is almost 15,000 individuals, while the fur seal *A. australis* constitutes the biggest colony in South America, with approximately 300,000 individuals (Páez 2000). The high mortality observed on the Uruguayan coast is comparable with that observed in Peru, Chile, and Argentina (Leguia et al. 2023; Plaza et al. 2024). Notably, sea lions were more affected than fur seals despite the latter’s larger population, suggesting a higher HPAIV H5N1 susceptibility.

Some reports have described HPAIV in marine mammals outside South America. The reported species included harbor seals (*Phoca vitulina*) and gray seals (*Halichoerus grypus*) in the northern hemisphere. Most of these cases were associated with pneumonia symptoms (Zohari et al. 2014; Bodewes et al. 2015; Gulyaeva et al. 2018), but recent reports show clinical neurological signs (Mirolo et al. 2023; Puryear et al. 2023) as observed in pinnipeds from Chile and Peru (Leguia et al. 2023; Ulloa et al. 2023). In Uruguay, respiratory symptoms were found in one sea lion, and neurological symptoms in one sea lion and three terns (Table 1). It is possible that some animals showed respiratory or neurological symptoms before deceased, but most were found dead without any clinical signs recorded.

The pinniped outbreak was associated with HPAIV H5N1 infection in four terns. Despite active surveillance between June and October 2023, these are the first HPAIV detections in wild birds in Uruguay. These seabirds inhabit coastal regions and share their habitat with marine mammals.

The avian influenza outbreak in pinnipeds and terns in Uruguay differs from that observed in wild birds and backyard poultry from February to May (Marandino et al. 2023). These outbreaks belong to separate phylogenetic groups, harbor-specific amino acid residues, and have non-overlapping tMRCA and ancestral hosts, supporting diverse geographic origins and a divergent transmission displacement (Fig. 1A and B, Supplementary Table S3). This indicates that Uruguay experienced two infection waves of HPAIV (H5N1) of clade 2.3.4.4b during 2023. The first wave, affecting wild birds and backyard poultry, arrived in Uruguay within weeks of detection on the Pacific coast of Chile and expanded over four months (February to May 2023). Outbreaks appeared interspersed in a major clade with strains from Chile and Argentina, indicating the possibility of multiple introductions (Marandino et al. 2023). Notably, a HPAIV (H5N1) strain of clade 2.3.4.4b from Brazil (May 2023) was genetically similar to the first-wave viruses, indicating the widespread distribution of this clade in South America (Reischak et al. 2023). The second monophyletic wave affects primarily pinnipeds and tern species, leading to an unusual mortality event in pinnipeds from September to October 2023. This latter behavior supports the idea that the second wave originates more recently from a common ancestor that entered the host population as a single event.

The second Uruguayan wave is characterized by strains harboring the 591K and 701N residues in the PB2 subunit of the RNA polymerase complex, a critical determinant of AIV host range and adaptation (Guilligay et al. 2008; Soh et al. 2019). The AIV polymerase performs poorly in mammalian cells, likely because its catalytic activity requires interaction with host proteins, such as importin-α and ANP32A, which differ between birds and mammals (Soh et al. 2019). Thus, to overcome host restriction of the polymerase, influenza virus strains in mammals usually acquire and select adaptive substitutions in PB2 (Mänz et al. 2016). Studies of natural zoonoses and experimental passaging have identified mutations that adapt polymerases to mammalian hosts, such as the detected Q591K and D701N substitutions associated with increased polymerase activity in mammalian cells (Chiang et al. 2016).

In the present analysis, PB2-591K and PB2-701N were restricted to clade 5 comprising three sea lions collected in March 2023 from Chile and Peru, a human case from Chile, and Uruguayan sea lions, fur seals, and terns (Fig. 1A and https://microreact.org/project/nSinj3LbMRme4gqussrTaU-influenzasea-lionurug). The phylogenetic similarity, the 591K/701N residues, and the ancestral state reconstruction suggest that Uruguayan viruses originated from similar strains circulating in pinnipeds from the Chilean and Peruvian coasts (Azat et al. 2023; Jimenez-Bluhm et al. 2023). The expansion of this successful lineage might be related to the combined residues that enhance the geographic spreading ability of the virus through mammalian hosts. Data from current and previous influenza global outbreaks indicate that the simultaneous occurrence of both mutations is rarely observed in nature (Pardo-Roa et al. 2023; Peacock et al. 2023). The 591K and 701N residues in our study might have coevolved through adaptive selection, facilitating replication in mammalian hosts (Peacock et al. 2023). Co-adaptation of PB2 mutations provides higher adaptability of AIVs in mammals and might contribute to crossing the interspecies barrier to mammals (Li et al. 2022).

Our findings have two remarkable implications uncommon for HPAIV epidemiology: the plausibility of mammal-to-mammal and mammal-to-bird transmissions.

Mammal-to-mammal transmission of HPAIV H5N1 is rare but has been proposed in captive tigers and experiments with cats (Thanawongnuwech et al. 2005; Rimmelzwaan et al. 2006). However, interspecific transmission from experimentally infected cats to naïve domestic dogs did not occur (Giese et al. 2008). Recent research also suggested mammal-to-mammal infection of clade 2.3.4.4b in fur farms (Agüero et al. 2023; Lindh et al. 2023).

Wild pinniped populations have peculiar characteristics like those observed in mammals kept in captivity. These highly social animals frequently interact through physical contact, fights, mate competition, and vocalizations such as spitting and hissing and have constant contact with the feces and urine of other colony animals. This interplay and the high-density populations may facilitate the transmission of HPAIV and serve as a model for studying mammal-to-mammal transmission in natural environments.

Influenza in pinnipeds spread throughout the coastline of Peru, Chile, Argentina, Uruguay, and recently Brazil (Adlhoch et al. 2023a; Plaza et al. 2024). This spread by proximity along the southern coast of South America, from the Pacific to the Atlantic Ocean (Supplementary Fig. S2), is consistent with the virus being transmitted mainly by pinnipeds. A primary mammal-to-mammal transmission also explains why the Uruguayan and Chilean strains of the virus are closely related, even though they were collected 6 months apart, and that many sea lions are currently affected simultaneously. We proposed that only the lineage harboring PB2-591K/701N mammalian adaptive residues expanded to Argentina, Uruguay, and Brazil. Unlike what has been observed in Chile and Peru, this expansion on the Atlantic coast was not preceded and concurrent with massive bird cases despite intensive surveillance (Supplementary Fig. S2) (Plaza et al. 2024).

The phylogenetic results and the 591K and 701N mammalian markers in tern viruses suggested that they have a mammalian origin and were directly acquired from sea lions and fur seals on the Uruguayan coasts. Pinnipeds spend time ashore in sites typically used by seabirds for roosting, facilitating cross-species transmission of AIV between these species.

Mammalian adaptive markers are linked with the passage of the virus through a mammalian host, and it is unlikely that they developed *de novo* in bird hosts. Moreover, as described in Chile, we have not observed intra-host variability (minor single-nucleotide variants) in the PB2-591/701 codons in marine mammals or avian genomes that support an ongoing change in mammalian adaptive residues (Supplementary Table S5) (Pardo-Roa et al. 2023). The ability of the virus to retain acquired genetic variability during the mammal passage is of significant concern since these viruses could be transmitted to other mammals, including humans, directly and potentially indirectly by affecting wild birds or poultry populations. Although these mammalian adaptations caused the death of some individuals (Table 1), there have not been massive death events registered in seabirds in the Uruguayan coastal region, suggesting that the virus has a lower spreading ability among birds.

The main limitation of our studies is the knowledge gap in the sequences available from some South American countries that affect the performance of phylogeographic inferences. Another concern is the lack of clinical data and the unavailability of tissue and blood samples to further the analysis.

Our study introduces a new perspective on influenza transmission in marine mammals in South America. It also offers an additional framework for understanding how avian influenza spreads and impacts bird and mammalian hosts. The challenge of controlling avian influenza is amplified by its spread across hosts adapted to air, water, and water–land interfaces. Viruses capable of infecting and replicating in pinnipeds may be more adapted to mammalian than avian hosts (Pardo-Roa et al. 2023). In addition to serving as a spillover host, other influenza subtypes could be endemic in Uruguayan pinnipeds (Arbiza et al. 2012), providing genetic reassortment opportunities. Lastly, the AIV interspecies transmission should be closely monitored through continued surveillance and containment measures to restrict transmission.

## Supplementary Material

veae031_Supp
