# Supplementary material for: Highly pathogenic avian influenza H5N1 virus infections in pinnipeds and seabirds in Uruguay: Implications for bird–mammal transmission in South America
Source: Virus Evol. 2024 Apr 13;10(1):veae031. doi: 10.1093/ve/veae031 (PMC11096771; doi:10.1093/ve/veae031)

Supplementary material

Tables and Figures

Highly pathogenic avian influenza H5N1 virus infections in pinnipeds and seabirds in Uruguay: implications for bird-mammal transmission in South America

Tomás et al. 2024

Table legends

Supplementary Table 1. Highly pathogenic **a**vian influenza H5N1 virus-positive cases in Uruguayan samples.

Supplementary Table 2. Coverage of the different highly pathogenic avian influenza virus H5N1 segments from Uruguayan strains.

Supplementary Table 3. Amino acid differences between highly pathogenic avian influenza H5N1 virus invasions in Uruguay at different times. Wave I (Feb-May 2023): wild birds and backyard poultry. Wave II (Sep-October 2023): pinnipeds and seabirds.

Supplementary Table 4. Amino acid changes between Uruguayan (wave II) and Chilean/Peruvian highly pathogenic avian influenza H5N1 virus strains.

Supplementary Table 5. Single nucleotide intra-host variant in Uruguayan strains of the highly pathogenic avian influenza H5N1 virus.

Supplementary Table 1. Highly pathogenic **a**vian influenza H5N1 virus-positive cases in Uruguayan samples.

Positive cases	Date*	Department	Location	Host class	Host species
1	2023 Sep 4	Montevideo	Playa del Cerro	Mammal	*Otaria flavescens*
2	2023 Sep 6	Maldonado	Punta del Este	Mammal	*Arctocephalus australis*
3	2023 Sep 6	Maldonado	Punta del Este	Mammal	*Otaria flavescens*
4	2023 Sep 6	Maldonado	Punta del Este	Mammal	*Otaria flavescens*
5	2023 Sep 6	Canelones	El Pinar	Mammal	Otariid
6	2023 Sep 6	Canelones	El Pinar	Mammal	Otariid
7	2023 Sep 6	Canelones	Lomas de Solymar	Mammal	Otariid
8	2023 Sep 8	Rocha	La Paloma	Mammal	*Arctocephalus australis*
9	2023 Sep 8	Rocha	Cabo Polonio	Mammal	*Otaria flavescens*
10	2023 Sep 8	Rocha	Cabo Polonio	Mammal	*Otaria flavescens*
11	2023 Sep 11	Canelones	Shangrilá	Mammal	Pinniped
12	2023 Sep 11	Canelones	Atlántida	Mammal	*Otaria flavescens*
13	2023 Sep 11	Canelones	Parque del Plata	Mammal	*Otaria flavescens*
14	2023 Sep 14	Canelones/Montevideo	Isla de Flores	Mammal	*Otaria flavescens*
15	2023 Sep 14	Canelones/Montevideo	Isla de Flores	Bird	*Sterna hirundinacea*
16	2023 Sep 14	Maldonado	Isla de Lobos	Mammal	Pinniped
17	2023 Sep 14	Maldonado	Isla de Lobos	Mammal	*Otaria flavescens*
18	2023 Sep 14	Rocha	Cabo Polonio	Mammal	Pinniped
19	2023 Sep 14	Rocha	Cabo Polonio	Mammal	Pinniped
20	2023 Sep 14	Rocha	Cabo Polonio	Mammal	Pinniped
21	2023 Sep 14	Rocha	Cabo Polonio	Mammal	Pinniped
22	2023 Sep 14	Rocha	Aguas Dulces	Mammal	*Otaria flavescens*
23	2023 Oct 2	Rocha	Cabo Polonio	Mammal	Pinniped
24	2023 Oct 2	Rocha	Cabo Polonio	Mammal	*Arctocephalus australis*
25	2023 Oct 2	Rocha	Cabo Polonio	Mammal	Pinniped
26	2023 Oct 2	Rocha	Cabo Polonio	Mammal	Pinniped
27	2023 Oct 2	Rocha	Cabo Polonio	Mammal	Pinniped
28	2023 Oct 2	Rocha	Cabo Polonio	Mammal	Pinniped
29	2023 Oct 2	Rocha	Cabo Polonio	Mammal	Pinniped
30	2023 Oct 2	Rocha	Cabo Polonio	Mammal	Pinniped
31	2023 Oct 4	Rocha	Cabo Polonio	Bird	*Sterna hirundinacea*
32	2023 Oct 4	Rocha	Cabo Polonio	Bird	*Sterna hirundinacea*
33	2023 Oct 4	Rocha	Cabo Polonio	Bird	*Sterna hirundinacea*

*Date of sample received.

SupplementaryTable 2. Coverage of the different highly pathogenic avian influenza virus H5N1 segments from Uruguayan strains.

Sample	Mean depth of coverage (Std Dev)	
P4_6923_*Otaria flavescens*		
PB2 gene	187.7 (31.4)[*1]	
PB1 gene	796.5 (619.8)[*1]	
PA gene	912.6 (124.0)[*1]	
HA gene	149.2 (149.8)[*1]	
NP gene	536.2 (66.2)[*1]	
NA gene	78.8 (91.5)[*1]	
M gene	361.2 (69.4)[*1]	
NS gene	272.5 (38.2)[*1]	
P5_6923_*Otaria flavescens*		
PB2 gene		16.6 (5.8)[*2]
PB1 gene		NC[†]
PA gene		848.8 (156.4)[*2]
HA gene		NC
NP gene		721.9 (96.9)[*2]
NA gene		545.1 (146.5)[*2]
M gene		1161.1(187.3)[*2]
NS gene		4004.8 (404.6)[*2]
P6_6923_*Otaria flavescens*		
PB2 gene		315.1 (69.9)[*2]
PB1 gene		NC
PA gene		1017.2 (185.8)[*2]
HA gene		174.6 (232.8)[*2]
NP gene		622.8 (106.7)[*2]
NA gene		836.2 (194.4)[*2]
M gene		2208.8 (368.5)[*2]
NS gene		2673.1 (268.9)[*2]
P7_6923_*Otaria flavescens*		
PB2 gene		121.6 (85.7)[*2]
PB1 gene		NC
PA gene		1237.7 (759.1)[*2]
HA gene		257.6 (303.5)[*2]
NP gene		339.0 (55.6)[*2]
NA gene		481.0 (124.1)[*2]
M gene		503.9 (84.5)[*2]
NS gene		2584.1 (258.7)[*2]
P8_8923_*Arctocephalus australis*		
PB2 gene	9.6 (3.4)[*1]	
PB1 gene	59.4 (12.4)[*1]	
PA gene	57.1 (10.9)[*1]	
HA gene	22.5 (5.9)[*1]	
NP gene	501.2 (81.5)[*1]	

NA gene	44.0 (10.5)*1	
M gene	5794.1 (1100.4)*1	
NS gene	2882.7 (324.4)*1	
P10_8923_*Otaria flavescens*		
PB2 gene	925.3 (128.7)*1	87.2 (28.4)*2
PB1 gene	1215.6 (370.5)*1	99.4 (34.8)*2
PA gene	1775.1 (212.4)*1	247.4 (33.8)*2
HA gene	5513.1 (3071.7)*1	481.7 (6533.9)*2
NP gene	7812.3 (794.9)*1	5371.4 (620.5)*2
NA gene	1898.5 (1139.5)*1	2872.2 (647.4)*2
M gene	32681.9 (5550.4)*1	1743.4 (367.7)*2
NS gene	9520.4 (1332.5)*1	2684.6 (560.1)*2
P13_11923_*Otaria flavescens*		
PB2 gene	434.0 (80.8)*1	
PB1 gene	798.4 (246.0)*1	
PA gene	964.2 (170.0)*1	
HA gene	917.4 (524.5)*1	
NP gene	1939.9 (228.1)*1	
NA gene	298.6 (254.2)*1	
M gene	3532.0 (612.2)*1	
NS gene	2435.1 (387.1)*1	
P14_11923_*Otaria flavescens*		
PB2 gene	35.1 (6.6)*1	2292.7 (305.0)*2
PB1 gene	66.4 (17.0)*1	6.4 (3.6)*2
PA gene	55.4 (12.8)*1	9412.5 (1039.2)*2
HA gene	58.5 (25.3)*1	146.0 (161.3)*2
NP gene	153.5 (20.1)*1	3747.5 (486.5)*2
NA gene	8.0 (5.3)*1	12336.0 (2400.7)*2
M gene	202.5 (38.4)*1	37618.0 (8382.3)*2
NS gene	71.1 (12.7)*1	6608.4 (1502.7)*2
P15_14923_*Otaria flavescens*		
PB2 gene		341.4 (340.7)*2
PB1 gene		NC
PA gene		NC
HA gene		225.4 (285.4)*2
NP gene		1812.2 (336.6)*2
NA gene		546.5 (137.7)*2
M gene		520.8 (88.4)*2
NS gene		2890.1 (343.0)*2
P16_14923_ *Sterna hirundinacea*		
PB2 gene	310.9 (82.7)*1	404.7 (149.6)*2
PB1 gene	647.3 (335.5)*1	48.7 (20.0)*2
PA gene	505.9 (93.4)*1	742.6 (123.9)*2
HA gene	3801.4 (2494.5)*1	15274.3 (8070.0)*2

NP gene	5165.8 (784.5)*[1]		14723.5 (1661.4)*[2]
NA gene	1370.1 (858.2)*[1]		3438.5 (535.3)*[2]
M gene	34512.2 (6018.9)*[1]		2215.8 (454.9)*[2]
NS gene	13722.1 (2212.0)*[1]		1433.6 (286.1)*[2]
P17_14923_*Otaria flavescens*			
PB2 gene	5.0 (2.3)*[1]		
PB1 gene	6.4 (3.3)*[1]		
PA gene	6.7 (2.7)*[1]		
HA gene	9.6 (3.5)*[1]		
NP gene	24.7 (7.2)*[1]		
NA gene	2.0 (1.2)*[1]		
M gene	36.0 (8.7)*[1]		
NS gene	23.4 (4.0)*[1]		
P18_14923_*Otaria flavescens*			
PB2 gene	20.4 (5.4)*[1]		2516.9 (354.9)*[2]
PB1 gene	18.5 (6.5)*[1]		10.5 (4.3)*[2]
PA gene	25.2 (4.6)*[1]		7948.3 (1024.8)*[2]
HA gene	47.4 (14.7)*[1]		497.0 (546.1)*[2]
NP gene	86.0 (16.1)*[1]		6739.5 (1064.4)*[2]
NA gene	19.9 (4.8)*[1]		15310.7 (3061.2)*[2]
M gene	231.9 (41.9)*[1]		29767.8 (6517.3)*[2]
NS gene	161.1 (31.0)*[1]		14944.5 (3311.9)*[2]
P23_41023_*Sterna hirundinacea*			
PB2 gene		745.0 (452.2)*[12]	
PB1 gene		32.7 (16.5)*[12]	
PA gene		1358.3 (397.9)*[12]	
HA gene		1256.8 (996.9)*[12]	
NP gene		2294.1 (371.8)*[12]	
NA gene		2226.3 (665.5)*[12]	
M gene		3414.6 (538.6)*[12]	
NS gene		10383.5 (904.9)*[12]	
P24_41023_*Sterna hirundinacea*			
PB2 gene		86.3 (17.8)*[12]	
PB1 gene		NC	
PA gene		130.2 (28.1)*[12]	
HA gene		115.5 (40.6)*[12]	
NP gene		1493.5 (223.9)*[12]	
NA gene		NC	
M gene		287.1 (59.5)*[12]	
NS gene		3666.9 (378.7)*[12]	
P25_41023_*Sterna hirundinacea*			
PB2 gene		2192.4 (796.3)*[12]	
PB1 gene		27.5 (55.5)*[12]	
PA gene		2204.2 (412.4)*[12]	

HA gene	333.7 (94.2)*[12]
NP gene	907.5 (128.7)*[12]
NA gene	94.4 (133.6)*[12]
M gene	6513.8 (1235.3)*[12]
NS gene	11985.2 (1131.6)*[12]
P26_21023_ *Arctocephalus australis*	
PB2 gene	888.5 (1341.7)*[12]
PB1 gene	NC
PA gene	1275.7 (1357.1)*[12]
HA gene	716.3 (888.0)*[12]
NP gene	1079.6 (177.0)*[12]
NA gene	999.4 (261.4)*[12]
M gene	2412.7 (360.2)*[12]
NS gene	8446.2 (756.8)*[12]

*[1]Library preparation from amplicons, according to Zhou et al. (2009).

*[2]Library preparation from amplicons, according to Hoffmann et al. (2001).

*[12]Library preparation from both PCR methods.
[†]Non-coverage.

Supplementary Table 3. Amino acid differences between highly pathogenic avian influenza H5N1 virus invasions in Uruguay at times. Wave I (Feb-May 2023): wild birds and backyard poultry. Wave II (Sep-October 2023): pinnipeds and different . seabirds

		PB2				PB1		PA					NP	NS1			
	Strain	122	154	**591***	**701***	515	646	20	57	86	237	548	119	21	26	53	226
Wave I	EPI_ISL_18310958	V	-	**Q**	**D**	S	I	A	R	M	E	M	I	R	E	D	I
	EPI_ISL_18310942	·	F	**·**	**·**	·	·	·	·	·	·	·	·	·	·	·	·
	EPI_ISL_18310967	·	·	**·**	**·**	·	·	·	·	·	·	·	·	·	·	·	·
	EPI_ISL_18310966	·	·	**·**	**·**	·	·	·	·	·	·	·	·	·	·	·	·
	EPI_ISL_18310965	·	·	**·**	**·**	·	·	·	·	·	·	·	·	·	·	·	·
	EPI_ISL_18310957	·	·	**·**	**·**	·	·	·	·	·	·	·	·	·	·	·	·
	EPI_ISL_18310964	·	·	**·**	**·**	·	·	·	·	·	·	·	·	·	·	·	·
	EPI_ISL_18310960	·	·	**·**	**·**	·	·	·	·	·	·	·	·	·	·	·	·
	EPI_ISL_18310959	·	·	**·**	**·**	·	·	·	·	·	·	·	·	·	·	·	·
	EPI_ISL_18310963	·	·	**·**	**·**	·	·	·	·	·	·	·	·	·	·	·	·
	EPI_ISL_18310962	·	·	**·**	**·**	·	·	·	·	·	·	·	·	·	·	·	·
	EPI_ISL_18310961	·	·	**·**	**·**	·	·	·	·	·	·	·	·	·	·	·	·
Wave II	P4_6923	I	L	**K**	**N**	A	M	T	Q	I	A	I	T	Q	K	G	T
	P5_6923	I	L	**K**	**N**	-	-	T	Q	I	A	I	T	Q	K	G	T
	P6_6923	I	L	**K**	**N**	-	-	T	Q	I	A	I	T	Q	K	G	T
	P7_6923	I	L	**K**	**N**	A	M	T	Q	I	A	I	T	Q	K	G	T
	P8_8923	I	L	**K**	**N**	A	M	T	Q	I	A	I	T	Q	K	G	T
	P10_8923	I	L	**K**	**N**	A	M	T	Q	I	A	I	T	Q	K	G	T
	P13_11923	I	L	**K**	**N**	A	M	T	Q	I	A	I	T	Q	K	G	T
	P14_11923	I	L	**K**	**N**	A	M	T	Q	I	A	I	T	Q	K	G	T
	P15_14923	I	L	**K**	**N**	A	M	T	-	-	-	-	T	Q	K	G	T
	P16_14923	I	L	**K**	**N**	A	M	T	Q	I	A	I	T	Q	K	C	T
	P17_14923	I	L	**K**	**N**	A	M	T	Q	I	A	I	T	Q	K	G	T
	P18_14923	I	L	**K**	**N**	A	M	T	Q	I	·	I	T	Q	K	G	T
	P23_41023	I	L	**K**	**N**	A	M	T	Q	I	A	I	T	Q	K	G	T
	P24_41023	I	L	**K**	**N**	A	M	T	Q	I	A	I	T	Q	K	G	T
	P25_41023	I	L	**K**	**N**	A	M	T	Q	I	A	I	T	Q	K	G	T
	P26_21023	I	L	**K**	**N**	A	M	T	Q	I	A	I	T	Q	K	G	T

*Markers associated with avian influenza virus adaptation to mammals.

Supplementary Table 4. Amino acid changes between Uruguayan (wave II) and Chilean/Peruvian highly pathogenic avian influenza H5N1 virus strains.

		PB2			PB1	PA					NP	NS1			
	Strain	122	**591***	**701***	515	20	57	86	237	548	119	21	26	53	226
Chile/Peru	EPI_ISL_18054503/dolphin/Peru	V	**Q**	**D**	S	A	R	M	E	M	I	R	E	D	I
	EPI_ISL_18054502/sea lion/Peru	·	·	·	·	·	·	I	·	·	·	·	·	·	·
	EPI_ISL_17805999/lion/Peru	·	·	·	·	·	·	·	·	·	·	·	·	·	·
	EPI_ISL_18054509/sea lion/Peru	·	·	·	·	·	Q	·	·	·	·	·	·	·	·
	EPI_ISL_17885978/sanderling/Chile	·	·	**N**	A	·	Q	·	·	·	T	·	K	G	T
	EPI_ISL_17885976/sea lion/Chile	·	·	**N**	A	·	Q	·	·	·	T	·	K	G	T
	EPI_ISL_17468386/human/Chile	·	**K**	**N**	·	T	Q	I	·	I	T	Q	K	G	T
	EPI_ISL_17777531/sea lion/Peru	·	**K**	**N**	A	·	Q	I	·	I	T	Q	K	G	T
	EPI_ISL_17777532/sea lion/Peru	·	**K**	**N**	A	·	Q	I	·	I	T	Q	K	G	T
	EPI_ISL_17885975/sea lion/Chile	·	**K**	**N**	·	·	Q	I	·	I	T	Q	K	G	T
Wave 2	P4_6923	I	**K**	**N**	A	T	Q	I	A	I	T	Q	K	G	T
	P5_6923	I	**K**	**N**	-	T	Q	I	A	I	T	Q	K	G	T
	P6_6923	I	**K**	**N**	-	T	Q	I	A	I	T	Q	K	G	T
	P7_6923	I	**K**	**N**	A	T	Q	I	A	I	T	Q	K	G	T
	P8_8923	I	**K**	**N**	A	T	Q	I	A	I	T	Q	K	G	T
	P10_8923	I	**K**	**N**	A	T	Q	I	A	I	T	Q	K	G	T
	P13_11923	I	**K**	**N**	A	T	Q	I	A	I	T	Q	K	G	T
	P14_11923	I	**K**	**N**	A	T	Q	I	A	I	T	Q	K	G	T
	P15_14923	I	**K**	**N**	A	T	-	-	-	-	T	Q	K	G	T
	P16_14923	I	**K**	**N**	A	T	Q	I	A	I	T	Q	K	C	T
	P17_14923	I	**K**	**N**	A	T	Q	I	A	I	T	Q	K	G	T
	P18_14923	I	**K**	**N**	A	T	Q	I	·	I	T	Q	K	G	T
	P23_41023	I	**K**	**N**	A	T	Q	I	A	I	T	Q	K	G	T
	P24_41023	I	**K**	**N**	A	T	Q	I	A	I	T	Q	K	G	T
	P25_41023	I	**K**	**N**	A	T	Q	I	A	I	T	Q	K	G	T
	P26_21023	I	**K**	**N**	A	T	Q	I	A	I	T	Q	K	G	T

*Markers associated with avian influenza virus adaptation to mammals.

Supplementary Table 5. Single nucleotide intra-host variant in Uruguayan strains of the highly pathogenic avian influenza H5N1 virus.

Sample/codon position	Change	Frequency (%)	Coverage	Polymorphism type
P5_6923_*Otaria flavescens*				
PA_147 codon	A > G	88 - 11.4	852	synonymous
NA_73 codon	T > A	88.4 - 11.6	859	synonymous
M_19 codon	C > T	88.7 - 10.9	1241	synonymous
M_158 codon	G > A	39.1 - 60.5	958	synonymous
NS1_66 codon	G > A	89.5 - 10.4	4460	synonymous
P6_6923_*Otaria flavescens*				
PB2_67 codon	A > G	87.2 - 12.8	290	I > V
NP_50 codon	A > G	62.9 - 36.8	587	S > G
P7_6923_*Otaria flavescens*				

NP_166 codon	G > T	78.8 - 20.9	392	synonymous
P8_8923_*Arctocephalus australis*				
M_82 codon	T > A	87.2 - 12.8	6475	N > K
P13_11923_*Otaria flavescens*				
PA_221 codon	G > A	88.9 - 11.1	835	synonymous
P15_14923_*Otaria flavescens*				
NP_104 codon	C > A	85.4 - 14.4	3512	Q > K
M_319 codon	C > A	55.4 - 44.4	597	H > N
NS1_36 codon	C > A	87.8 - 12.1	3006	synonymous
NS1_122 codon	C > A	85.6 - 14.4	2566	synonymous
P23_41023_*Sterna hirundinacea*				
NP_375 codon	G > A	82.3 - 17.5	2246	D > N
NA_169 codon	C > T	89.1 - 10.7	1603	P > S
P25_41023_*Sterna hirundinacea*				
PB2_136 codon	G > A	76 - 23.6	1507	R > K
PB2_545 codon	G > A	75.6 - 24.3	1682	synonymous
HA_517 codon	A > G	78.3 - 21.5	447	synonymous
NP_223 codon	T > C	85 - 14.7	822	synonymous
P26_21023_ *Arctocephalus australis*				
PB2_13 codon	G > A	81.2 - 18.8	6142	synonymous
PA_658 codon	T > G	86.8 - 13.1	2761	F > L
NP_422 codon	A > G	82.1 - 17.9	944	synonymous
M_186 codon	C > T	70.9 – 29	2123	synonymous
M_237 codon	G > A	84.8 - 15.1	1776	synonymous
M_319 codon	C > A	87.7 - 12.2	2591	H > N

Figure legends

Supplementary Figure 1. Phylogenetic analysis of the highly pathogenic avian influenza H5N1 virus coding regions from South America. Uruguayan strains are highlighted in light brown (Wave I) and light blue (Wave II). Hosts and mammalian adaptive residues (PB2-591Q/701N) are depicted. A) Maximum-likelihood tree based on segment 1 (PB2); B) Maximum-likelihood tree based on segment 2 (PB1); C) Maximum-likelihood tree based on segment 3 (PA); D) Maximum-likelihood tree based on segment 4 (HA); E) Maximum-likelihood tree based on the segment 5 (NP); F) Maximum-likelihood tree based on the segment 6 (NA); G) Maximum-likelihood tree based on the segment 7 (M); H) Maximum-likelihood tree based on the segment 8 (NS).

Supplementary Figure 2: Variation in the distribution of the highly pathogenic avian influenza H5N1 avian influenza virus in South America (Microreact instance including all confirmed cases cataloged by FAO in South America after January 1st, 2023). A) Cases until 31 July 2023. Registers showed an initial coastal and an internal spreading involving wild birds, backyard poultry, and outbreaks in commercial farms. B) Cases from 1st August to October 2023. Registers appeared south of the Atlantic coast and spread northward, with fewer cases involving birds and case detections in the interior of South America.

Supplementary Figure 1. Phylogenetic analysis of the highly pathogenic avian influenza H5N1 virus coding regions from South America. Uruguayan strains are highlighted in light brown (Wave I) and light blue (Wave II). Hosts and mammalian adaptive residues (PB2-591Q/701N) are depicted. A) Maximum-likelihood tree based on segment 1 (PB2); B) Maximum-likelihood tree based on segment 2 (PB1); C) Maximum-likelihood tree based on segment 3 (PA); D) Maximum-likelihood tree based on segment 4 (HA); E) Maximum-likelihood tree based on the segment 5 (NP); F) Maximum-likelihood tree based on the segment 6 (NA); G) Maximum-likelihood tree based on the segment 7 (M); H) Maximum-likelihood tree based on the segment 8 (NS).

Supplementary Figure 1A (segment 1 PB2 gene)

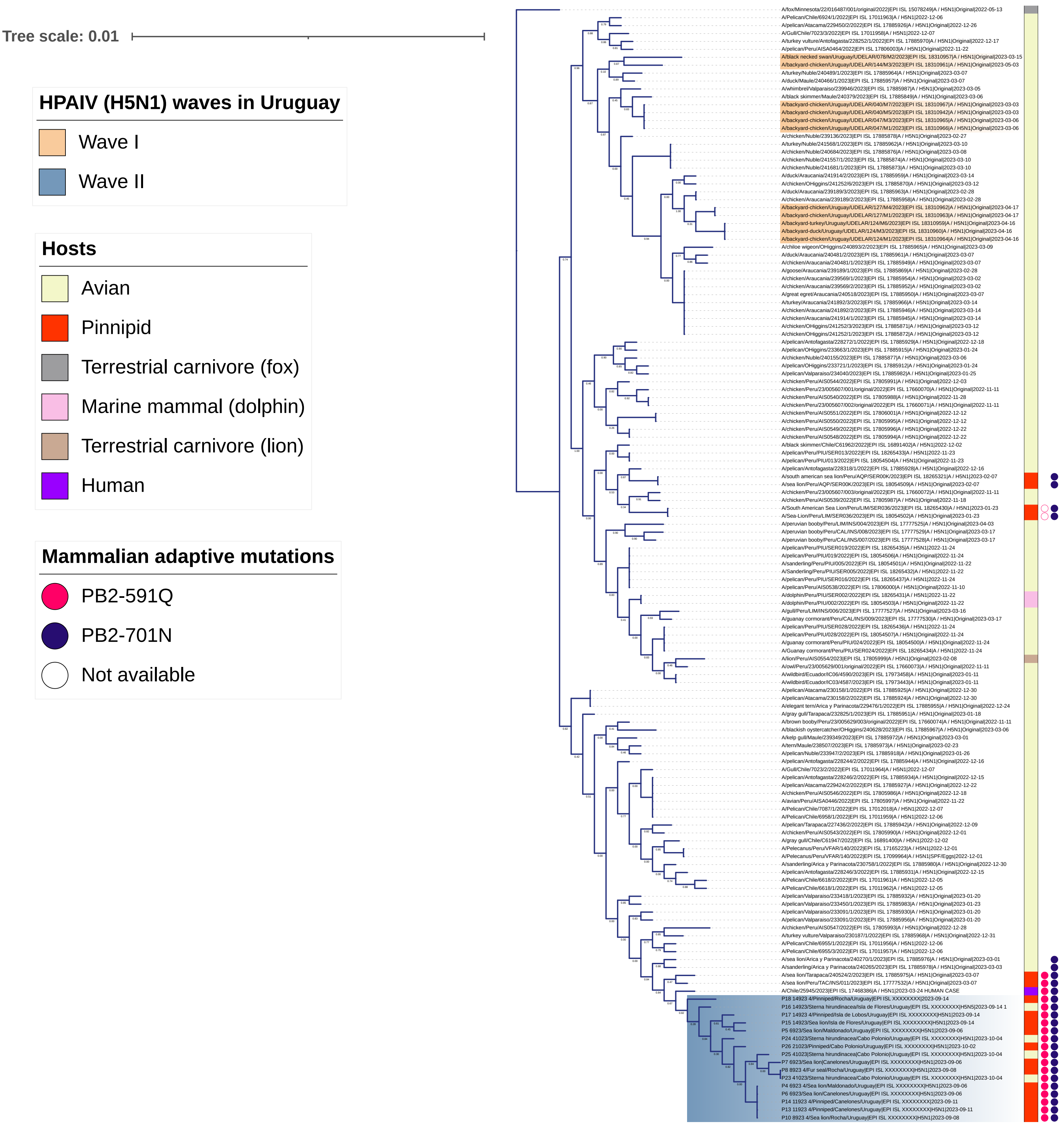

Supplementary Figure 1B (segment 2 PB1 gene)

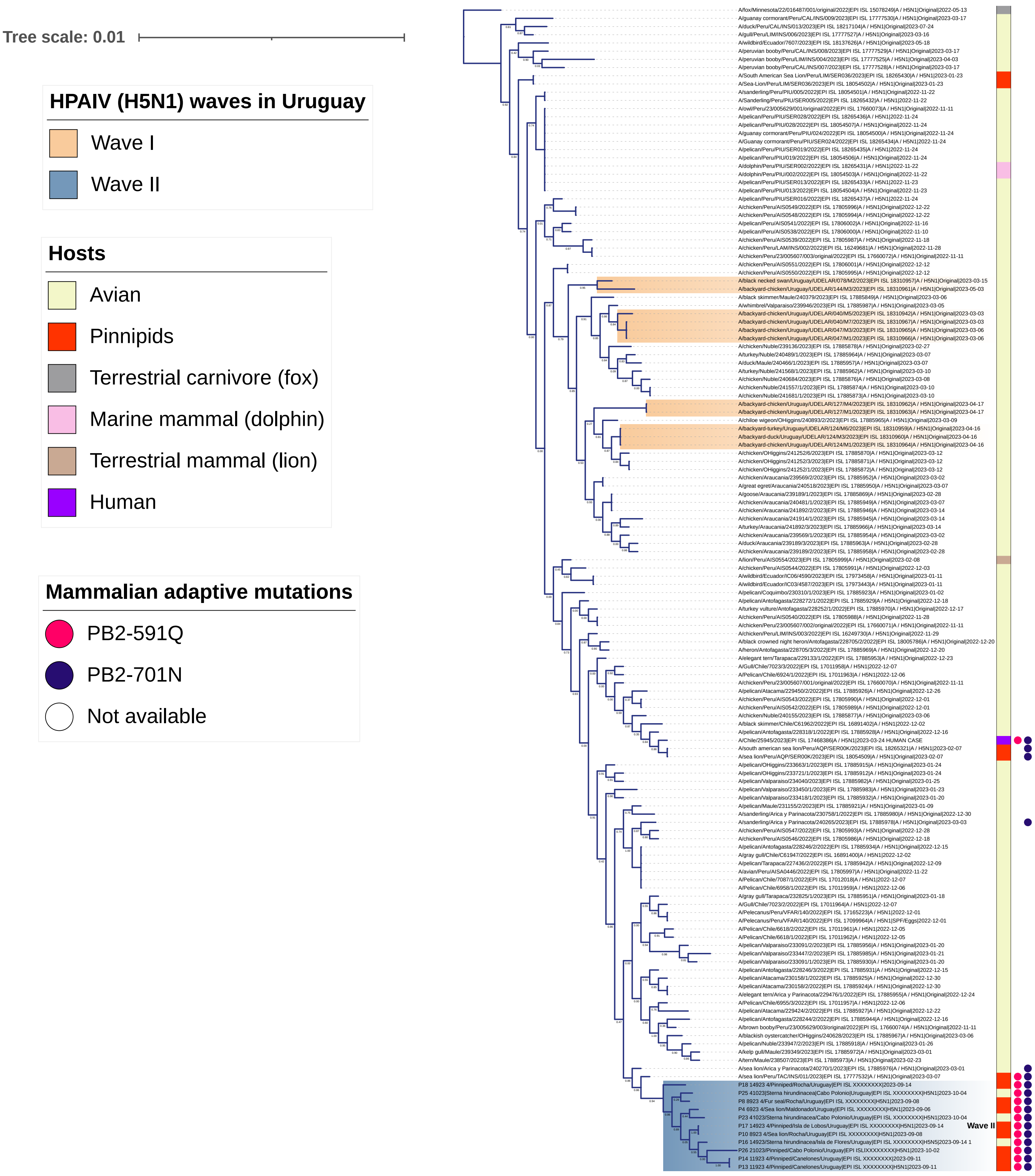

Supplementary Figure 1C (segment 3 PA gene)

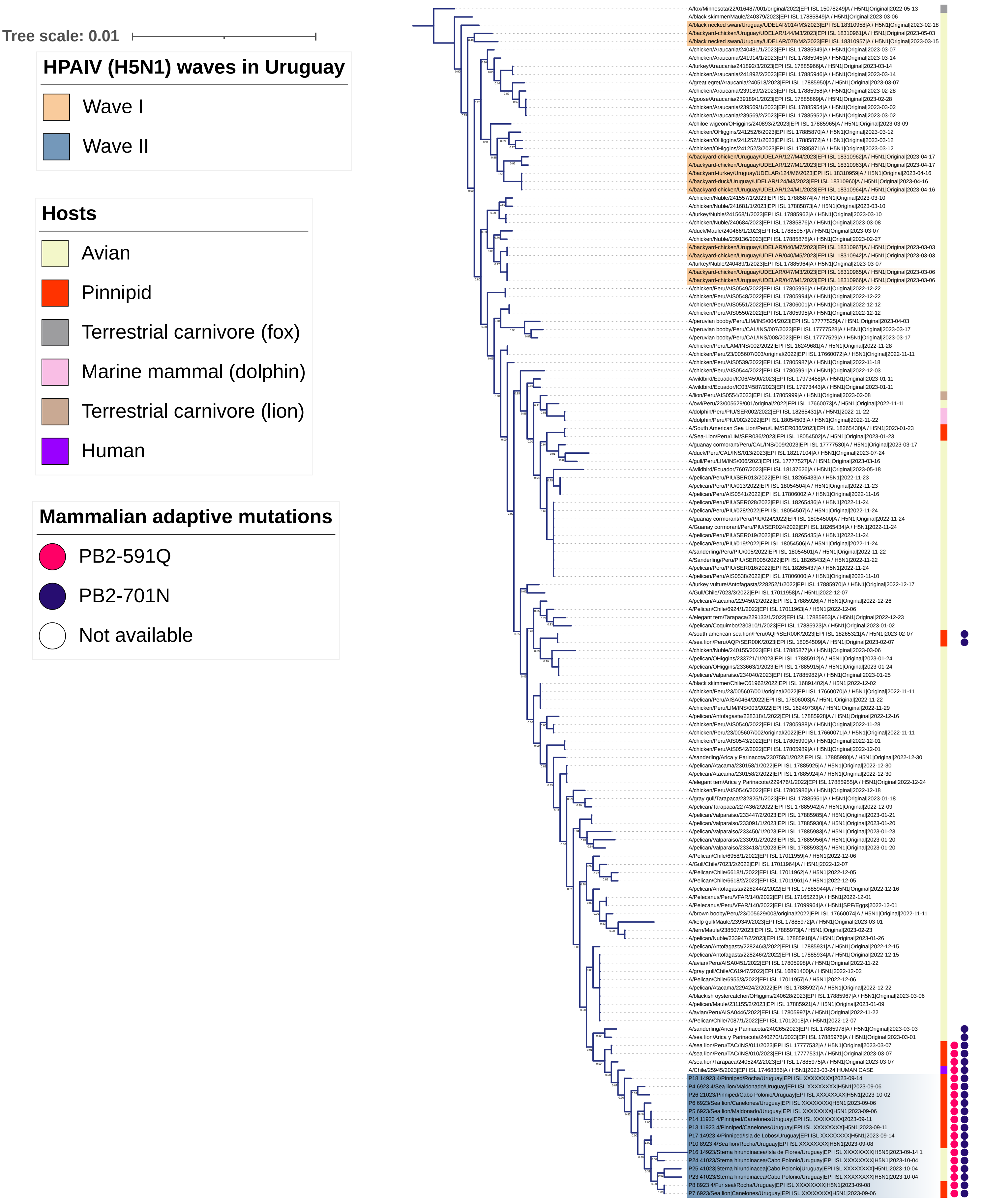

Supplementary Figure 1D (segment 4 HA gene)

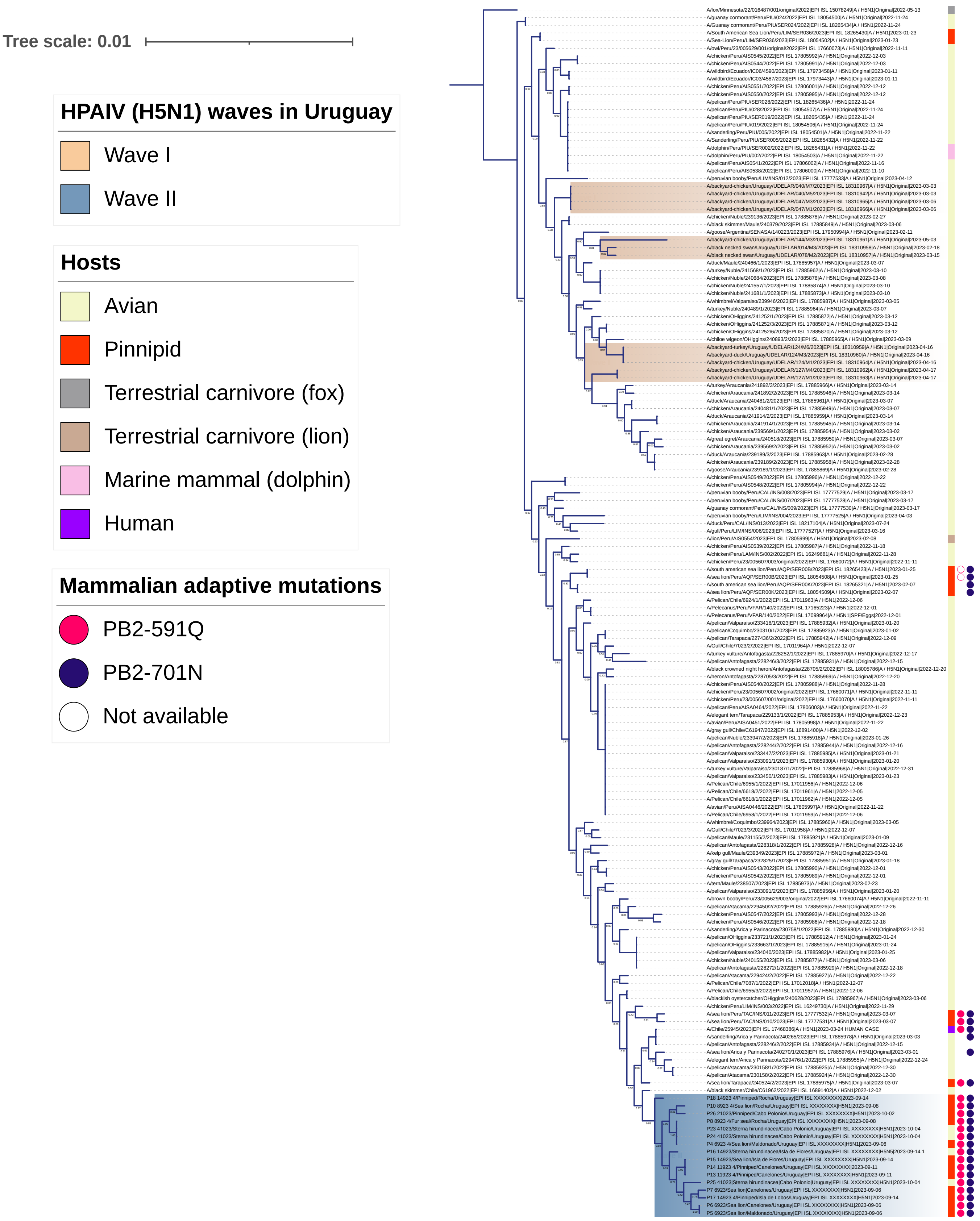

Supplementary Figure 1E (segment 5 NP gene)

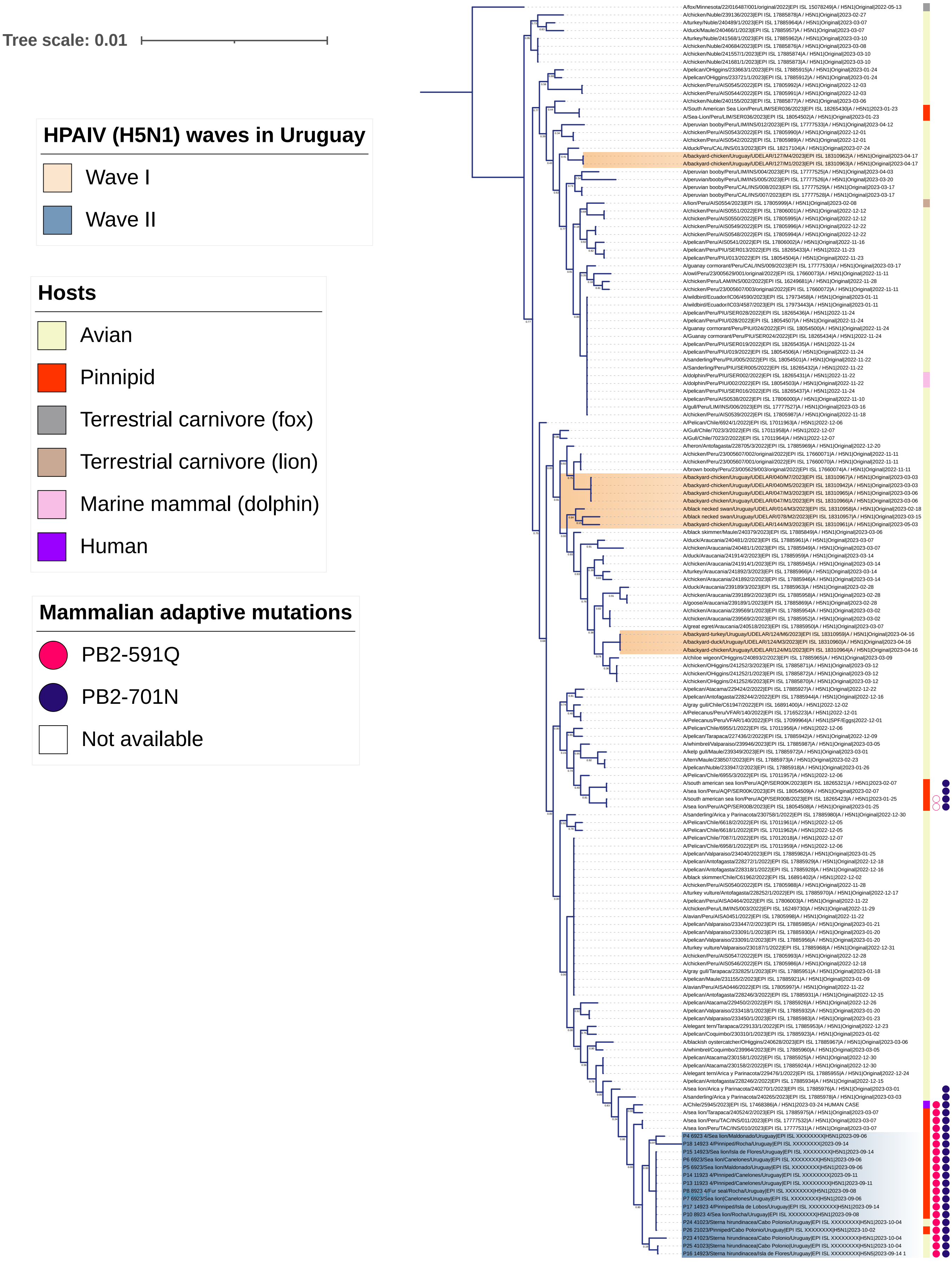

Supplementary Figure 1F (segment 6 NA gene)

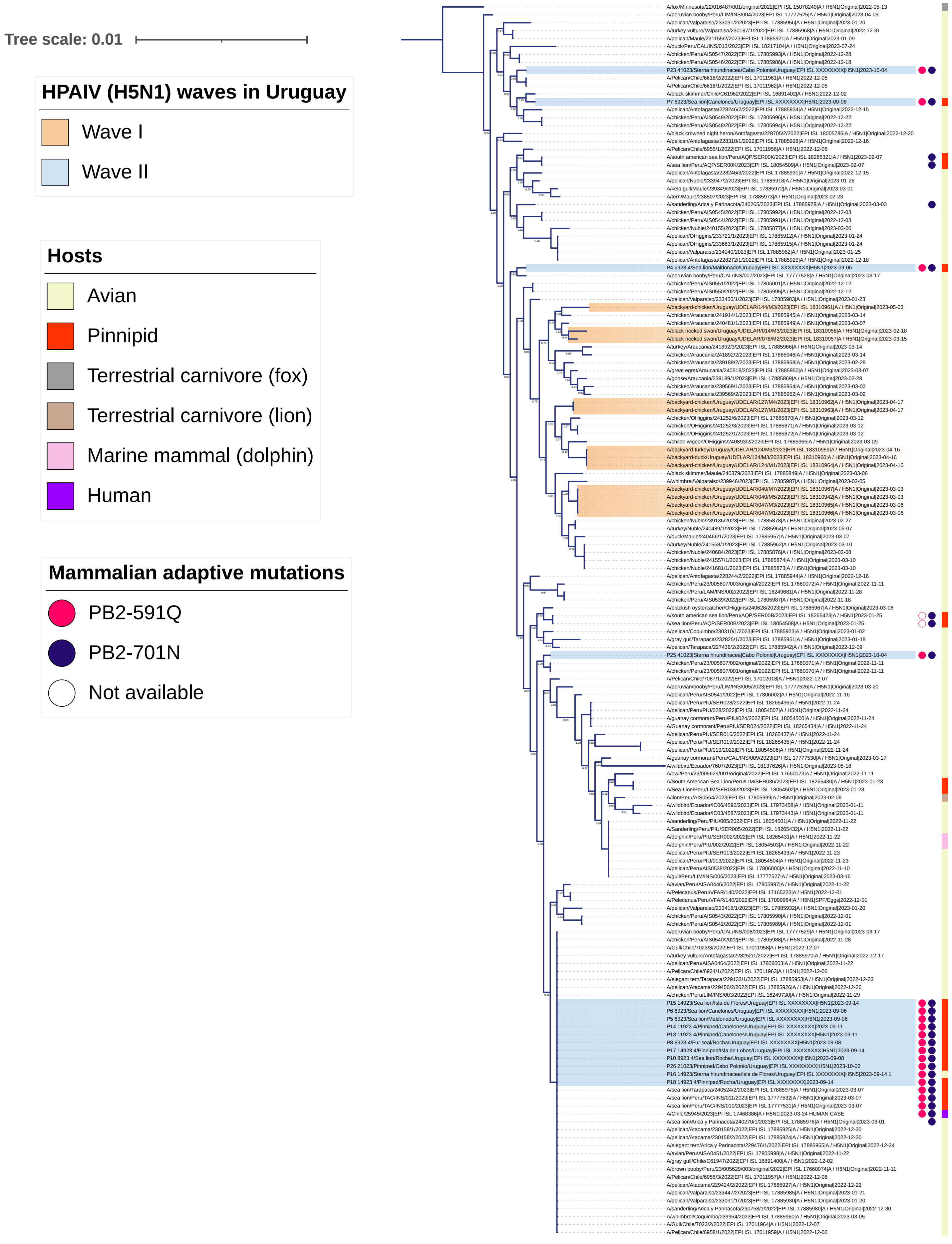

Supplementary Figure 1G (segment 7 M gene)

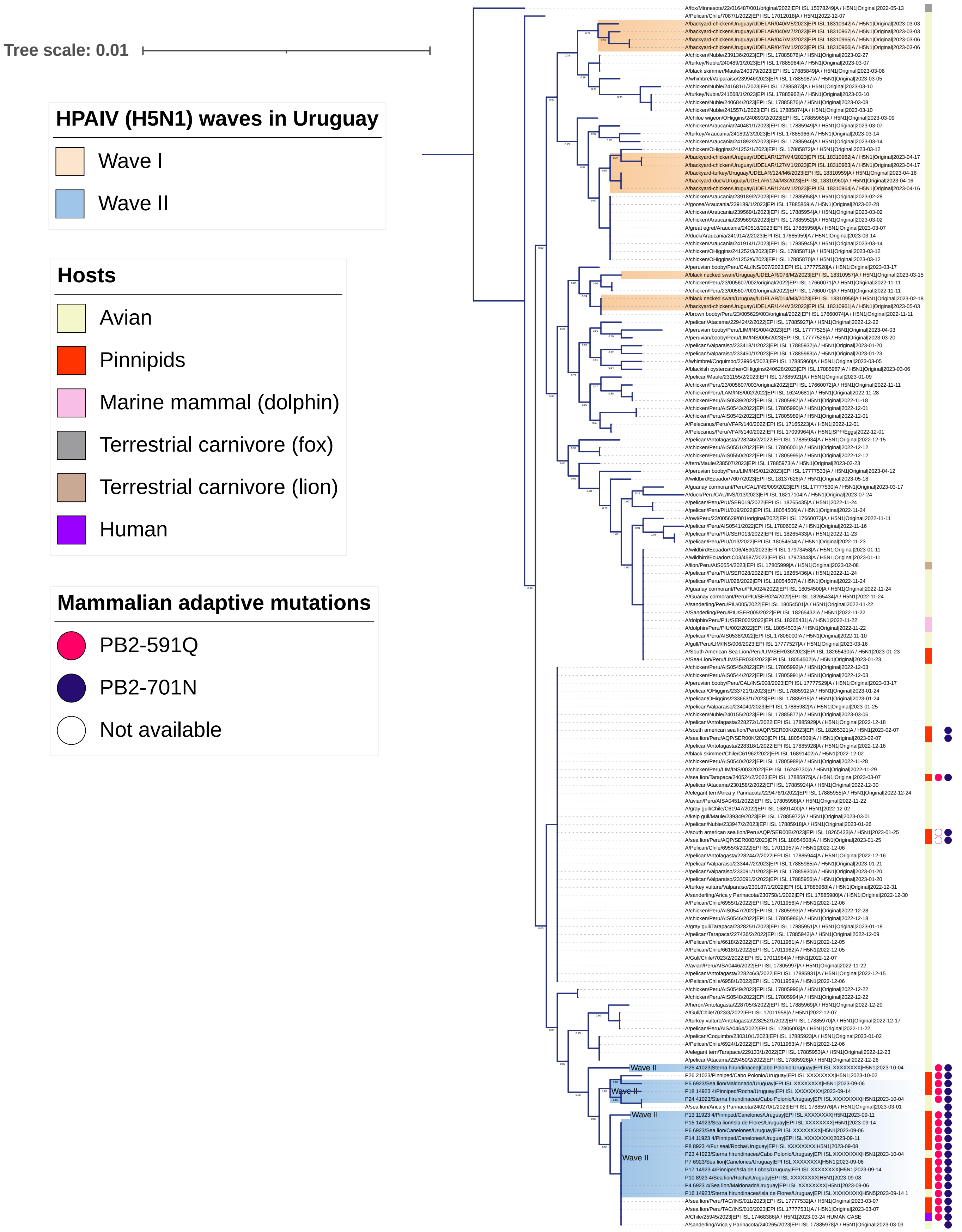

Supplementary Figure 1H (segment 8 NS gene)

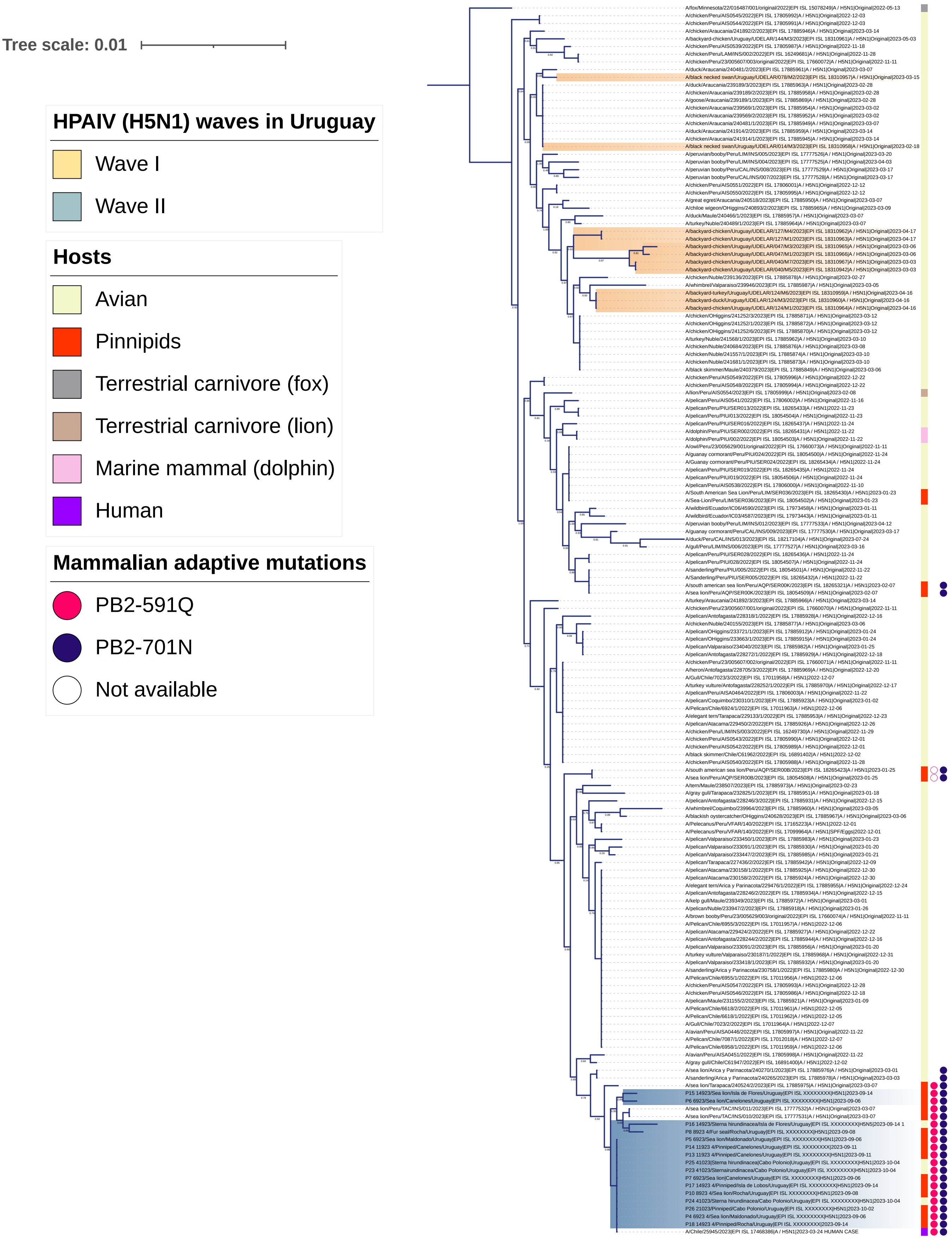

Supplementary Figure 2: Variation in the distribution of the highly pathogenic avian influenza H5N1 avian influenza virus in South America (Microreact instance including all confirmed cases cataloged by FAO in South America after January 1st, 2023). A) Cases until 31 July 2023. Registers showed an initial coastal and an internal spreading involving wild birds, backyard poultry, and outbreaks in commercial farms. B) Cases from 1st August to October 2023. Registers appeared south of the Atlantic coast and spread northward, with fewer cases involving birds and case detections in the interior of South America.

Supplementary Figure 2

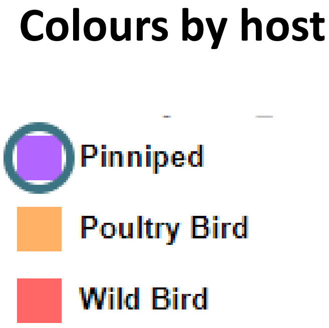

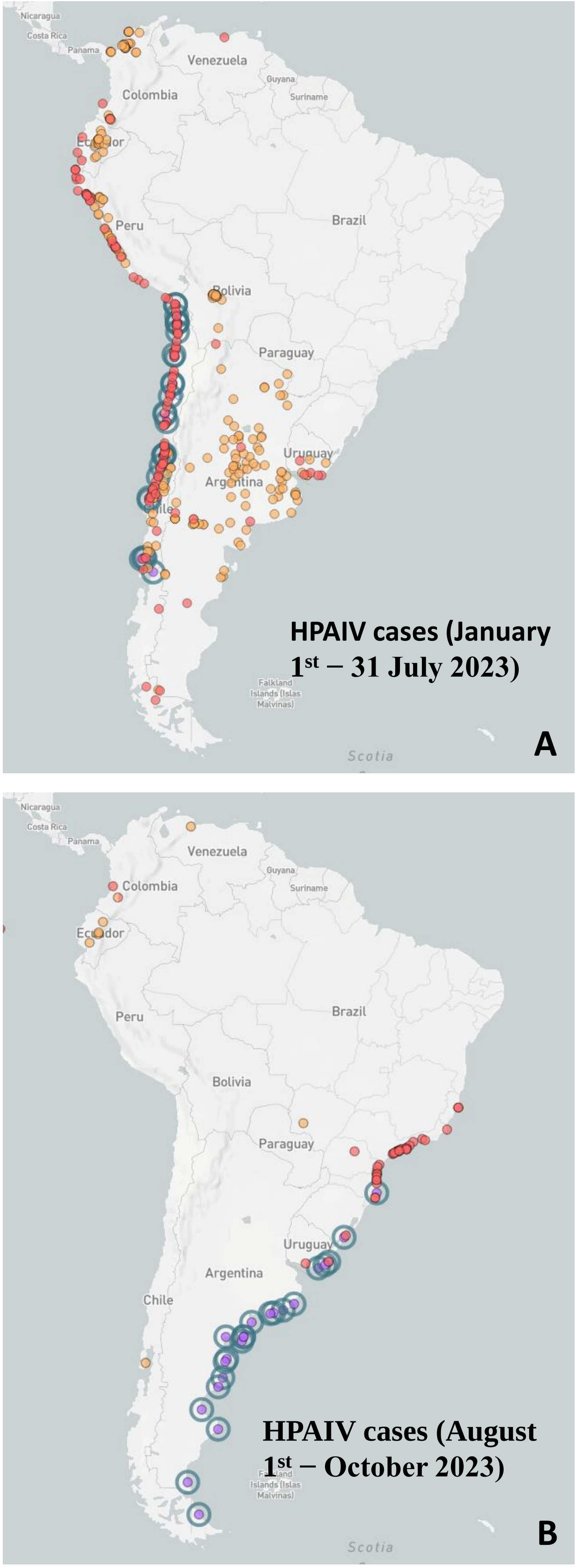

Supplement: veae031_Supp [file veae031_supp.zip › suppl_data/Supplementary material.pdf]
